# The Lübeck Medication Satisfaction Questionnaire—A Novel Measurement Tool for Therapy Satisfaction

**DOI:** 10.3390/jpm13030505

**Published:** 2023-03-10

**Authors:** Ludwig Matrisch, Yannick Rau, Hendrik Karsten, Hanna Graßhoff, Gabriela Riemekasten

**Affiliations:** 1 Department of Rheumatology and Clinical Immunology, Universität zu Lübeck, 23562 Lübeck, Germany; 2 Faculty of Medicine, Universität zu Lübeck, 23562 Lübeck, Germany; 3 BG Klinikum Hamburg, 21033 Hamburg, Germany; 4 Medical Faculty, University of Hamburg, 20251 Hamburg, Germany

**Keywords:** therapy satisfaction, treatment satisfaction, medication satisfaction, patient-reported outcomes

## Abstract

Background: Therapy satisfaction is widely considered an important aspect of clinical care. Still, there are currently no freely available questionnaires for its measurement. We developed the Lübeck Medication Satisfaction Questionnaire (LMSQ) for that purpose. Here, we present its content and psychometric properties. Methods: The LMSQ was validated on 86 patients in a single center study. The Kaiser-Meyer-Olkin test, confirmatory factor analysis, covariance analysis, and a test of exact fit were performed. Reliability was tested using Cronbach’s α and McDonald’s ω. The relationship to other patient-reported outcomes was tested using Pearson’s correlation. Results: Confirmatory factors analysis yielded moderate factor loadings with *p* < 0.001 in all subscales. Reliability was adequate (α = 0.857 and ω = 0.872). Model fitness was excellent in all tests. The LMSQ was positively correlated with medication adherence (r = 0.603, *p* < 0.001) and most dimensions of health literacy. Conclusions: The LMSQ possesses adequate psychometric properties for its purpose. We recommend further validation in a more diverse patient collective.

## 1. Introduction

We currently live in an era of increasingly personalized medical care [1]. The term refers to a philosophy of healthcare in which healthcare professionals take characteristics unique to their patients into account. This often refers to individualized therapy approaches guided by precise diagnostics on a molecular level [2]. However, personalized medical care can also take the patients’ values, goals, and their personal life situation into account [3]. This creates the basis for a trustful doctor-patient relationship. The quality of this relationship is associated with improved medication adherence and thereby improved therapy outcome [4].

Therapy satisfaction is defined as the degree to which the patients perceive that the treatment fulfills their health needs [5]. On one hand, therapy satisfaction can strengthen the trust between a patient and their healthcare providers [6], which can possibly lead to better health outcomes [7]. On the other hand, good therapy outcome can improve therapy satisfaction [8].

Therefore, improving therapy satisfaction is widely considered an important goal in healthcare [9]. Despite this importance, there is a lack of tools for the quantification of therapy satisfaction. The currently most widely used questionnaire—the Treatment Satisfaction Questionnaire for Medication (TSQM)—is a well validated tool available in several versions, but it lacks accessibility due to its licensing structure [10,11,12]. Healthcare providers or researchers in developing countries lacking sufficient funds might be unable to pay for it, hindering research as well as individualized treatment strategies.

This lack of adequate diagnostic tools for therapy satisfaction reflects the minor role it has played in the concept of personalized medicine so far. In psychology, it is well known that the experiences that shape the traits, values, and personal preferences are unique to the individual [13,14,15]. Taking those experiences into consideration therefore reflects the approach and aspiration of personalized medicine. To fulfill this task, there is a need for further research in that field as well as for a cultural shift in clinical practice.

### 1.1. Measurement of Therapy Satisfaction

Therapy satisfaction is a highly individual and subjective modality. Therefore, it is impossible to measure objectively [16]. Researchers as well as healthcare providers have to rely on patients’ reports. This can be performed in the form of an interview. Although interviews are an essential tool in qualitative research, they often lack the properties needed for quantitative analysis [17]. Furthermore, they rely on trained interviewers to minimize interviewer bias and thereby bind personnel capacities that often are not available in everyday clinical practice [18]. Therefore, self-reported questionnaires are often used to address these issues. Well-designed questionnaires give the opportunity to not only quickly and accurately quantify self-reported patient outcomes but to also assess multiple dimensions of said outcomes. Our goal was to develop an easily applicable questionnaire for the quantification of therapy satisfaction that could accurately differentiate between its dimensions.

### 1.2. The Lübeck Medication Satisfaction Questionnaire

The Lübeck Medication Satisfaction Questionnaire (LMSQ) is a self-administered questionnaire consisting of 18 statements concerning patients’ satisfaction with their medication. The patients can state their degree of agreement on a four-point Likert scale. The questionnaire was developed after an extensive literature review on therapy satisfaction and the factors influencing it. Adequate phrasing and intelligibility were ensured through a pilot survey consisting of a series of patient interviews as well as expert interviews before the study. The questionnaire consists of six subscales reflecting the dimensions of therapy satisfaction. Each subscale is measured by three equal-weighted statements in the questionnaire. The full questionnaire is presented in Table 1. The German version of the LMSQ is presented in Supplementary Table S1.

### 1.3. Evaluation of the LMSQ

Each item of the questionnaire can be assigned to a subscale. Every subscale consists of three items. The score of each subscale is calculated by adding up the scores of the three items and dividing them by three.

The subscales include the following:

Side effects (LMSQ_2, LMSQ_9, LMSQ_17): This subscale describes the patients’ degree of satisfaction with the side effects of their treatment. It is well known that side effects affect therapy satisfaction as well as other factors such as quality of life (QOL), medication adherence, and treatment outcome [19,20,21]. Therefore, side effects should be duly considered when deciding between therapy options. This subscale, however, does not measure the objective degree of side effects since it is a patient-reported outcome (PRO). Rather, the perceived subjective burden of side effects the patients experience is quantified.

Effectivity (LMSQ_5, LMSQ_11, LMSQ_14): This subscale describes the perceived effectivity of the therapy. Treatment effectivity plays an important role in the choice of therapy and therefore is the parameter usually measured in clinical studies. However, this section also does not measure the actual effect of the therapy but rather the effect as it is perceived by the patients.

Practicability (LMSQ_1, LMSQ_3, LMSQ_7): This subscale describes the practicability of the therapy. This is an important dimension of therapy satisfaction that deals with the non-biological properties of the therapy relevant to the patients. It incorporates how well the therapy resonates with the patients’ daily life schedule and their personal preferences. These factors have important implications for outcomes such as medication adherence and should be considered in therapy decisions. This is especially important from a perspective of personalized medicine.

Daily life (LMSQ_10, LMSQ_12, LMSQ_16): This subscale describes the patients’ degree of satisfaction with the freedom and independence gained through the therapy. Disease burden is an important factor influencing patients’ QOL [22]. Its alleviation can create QOL improvement for patients. Therefore, it is an important dimension of therapy satisfaction.

Healthcare workers (LMSQ_6, LMSQ_8, LMSQ_13): This subscale describes the patients’ degree of satisfaction with their healthcare providers. The quality of the relationship between patients and their healthcare providers has been shown to be of importance for therapy satisfaction as well as therapy outcome [23,24]. The style of communication as well as taking the patients’ values and goals into account is crucial for forming effective patient-healthcare worker relationships [25]. This should be a foremost goal in personalized therapy.

General satisfaction (LMSQ_4, LMSQ_15, LMSQ_18): This subscale describes the overall therapy satisfaction of the patient. This section is especially important from a personalized medicine perspective as it reflects the balance of factors influencing therapy satisfaction individually.

The total LMSQ score is calculated by adding up the scores of the individual items and dividing them by 18.

## 2. Materials and Methods

### 2.1. Patient Recruitment

The LMSQ was validated in its German version within a study investigating medication adherence and its influencing factors in patients with systemic sclerosis (SSc) [26]. A total of 88 patients with SSc were enrolled in a cross-sectional study at the Department of Rheumatology and Clinical Immunology at the University of Lübeck, Germany. Two patients dropped out due to being released from the hospital before they could finish the questionnaire, resulting in a dropout rate of 2.27%. Patients were recruited in the weekly SSc outpatient clinic as well as in the ward. Data were collected between July 2020 and February 2021. Patients fulfilling the EULAR/ACR 2016 classification criteria currently under treatment for their SSc were included [27]. We excluded patients unable to complete the questionnaire due to physical impairment, language barrier or illiteracy, as well as patients who legally could not consent to the study. This involved two patients, one who did not understand German and one who was physically unable to hold a pen. All other patients were asked to participate and left alone for the completion of the questionnaire to eliminate possible bias due to the Hawthorne effect. Anonymization was ensured by assigning a number to the participants.

**Figure 1:**
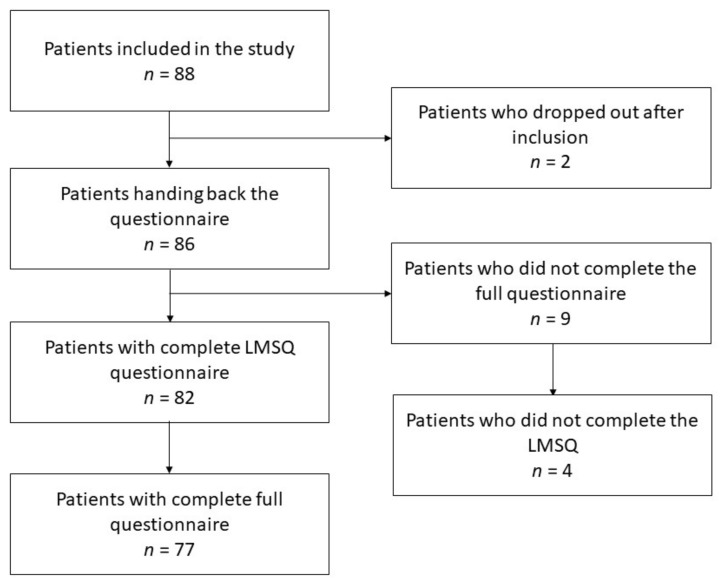
Recruitment process.

Additionally to the LMSQ, patients were also asked to provide information about their age, gender, native language, their migration background, their religion, their highest educational degree, their current employment status, and the number of members in their household for demographic purposes. The Scleroderma Health Assessment Questionnaire (SHAQ), the Compliance Questionnaire of Rheumatology (CQR), and the Health Literacy Questionnaire were also applied to assess associations between these patient-reported outcomes [28,29,30]. Nine patients did not fully complete their whole questionnaires, and out of those, four did not fully complete the LMSQ section of the full questionnaire. The patient recruitment process is illustrated in Figure 1. Since the various sections of the questionnaires could be evaluated independently, it was decided to include all participating patients, irrespective of the completeness for all clinical data.

### 2.2. Translation and Cultural Adaption into English

Since the questionnaire was initially created as well as used in this study in the German language, we translated it to make it more accessible to the international research and healthcare community. The translation process was led by the guidelines proposed by Beaton et al. [31]. In the first step, two translators were asked to independently translate the original version into English. The two translations were merged into one combined version which was translated back by a third translator into German in the second step. In the third step, the back-translated version was compared to the original version.

The German version that was used in this study is accessible in the supplement of this article.

### 2.3. Statistical Analysis

Statistical analysis was performed using Jamovi 1.2.27.0. Sampling adequacy was assessed using the Kaiser-Meyer-Olkin (KMO) test [32]. The cut-off value for insufficient sampling was set at 0.6. Confirmatory factor analysis was performed for the subscales of the LMSQ. Beforehand, we performed Bartlett’s test of sphericity. Reliability was analyzed using Cronbach’s α and McDonald’s ω. Pearson’s correlation was performed to elucidate the relationship between the PROs. Furthermore, we conducted an analysis of the factor covariances and performed a test of exact fit.

The significance level was set at *p* = 0.05.

## 3. Results

### 3.1. Demographic Characteristics of the Participants

Table 2 presents the demographic characteristics of the enrolled participants. All patients were treated for SSc. Miscellaneous clinical characteristics are not presented here as they are of less importance for the study of therapy satisfaction [33]. The patients are representative of SSc patients in Germany. The collective mainly consisted of women (75.3%), and very young and very old patients are represented less than proportionally. Additionally, the number of retirees was higher than in the general population (54.1%). Apart from these factors, the collective is representative of the general German population.

### 3.2. Descriptives of the LMSQ and Its Subscales

Table 3 features the descriptive statistics of the LMSQ. Four out of the 86 participants did not fill out the questionnaire completely due to unknown reasons, resulting in a response rate of 95.35%. The average and the median values are similar across all the subscales as well as the total LMSQ score.

### 3.3. Kaiser-Meyer-Olkin Test Revealed Sufficient Sampling Adequacy

To test the statistical requirements of the factor analysis, Bartlett’s test of sphericity and the Kaiser–Meyer–Olkin (KMO) test were performed. Bartlett’s test (χ² = 599, df = 153, *p* < 0.001) was significant, hinting towards a statistically significant difference between the correlation matrix and the identity matrix. The KMO test’s overall measure of sampling adequacy (MSA) was 0.811. Across all items of the LMSQ, the MSA was >0.6. Thus, we considered the MSA to be meritorious and the prerequisites to perform the factor analysis to be established. The full KMO test results are presented in Table 4.

### 3.4. LMSQ Is Characterized by Adequate Internal Validity and Reliability

A confirmatory factor analysis was performed for the subscales of the LMSQ presenting the dimensions of therapy satisfaction. The results are presented in Table 5. The factor loadings of all LMSQ items are significant (*p* < 0.001). The factor loadings range from 0.318 (LMSQ_1 and LMSQ_18) to 0.581 (LMSQ_5).

A test for exact fit showed adequate model fitness (χ² = 163, df = 120, *p* = 0.005). Additional fit measures are presented in Table 6. These additional measures of fitness further underscore the adequacy of our model.

An analysis of the factor covariances is presented in Table 7. Factor variances are moderate across the subscales of the LMSQ. Significant results were seen along most of the subscales. However, the covariances between side effects and daily life (estimate = 0.257, *p* = 0.09), practicability and daily life (estimate = 0.151, *p* = 0.35) and practicability and healthcare workers (estimate = 0.179, *p* = 0.25) were not significant.

To assess the scale reliability of the LMSQ, internal consistency was tested using Cronbach’s α as well as McDonald’s ω. Both proved adequate reliability of the LMSQ items (0.857 and 0.872, respectively).

### 3.5. Total LMSQ Score Correlated with Therapy Adherence and Health Literacy Assessed by CQR and HLQ Score

The LMSQ score correlated with therapy adherence measured by the CQR score (Pearson’s r = 0.566, *p* < 0.001). Controlling for age, SHAQ score, and disease duration, the correlation was r = 0.603, *p* < 0.001.

Pearson’s correlation with the subscores of the HLQ controlling for age, SHAQ score, and time since the disease onset yielded the results presented in Table 8. LMSQ correlated with all subscores of the HLQ besides appraisal.

## 4. Discussion

### 4.1. LMSQ Compared to Other Tools Assessing Therapy Satisfaction

The LMSQ is a novel tool for the measurement of therapy satisfaction in patients with systemic sclerosis. It was developed by applying rigorous scientific evaluation and showed excellent reliability and validity in the data presented in this article. It was developed for application in long-term drug therapy in chronic diseases. However, in its current state of validation, it has only been applied on patients with SSc. The reliability and validity for the therapy of acute medical issues or non-drug interventions such as surgery has not been tested.

The LMSQ is designed to distinguish between six dimensions of therapy satisfaction. This provides a more detailed and differentiated insight into patient views compared to already used questionnaires such as the TSQM or the Treatment Satisfaction with Medicines Questionnaire (SATMED-Q), which feature less dimensions [10,34]. The three questionnaires all feature a scale for the effectiveness of the treatment, for side effects, and for the general satisfaction with the treatment. The LMSQ as well as the SATMED-Q additionally feature a scale that represents the satisfaction with the medical care provided by the healthcare workers. While the TSQM and the SATMED both feature one scale to assess the convenience of the treatment, this property is split up into two scales (practicability and daily life) in the LMSQ to differentiate between the practicability of the treatment and its impact on the patients’ daily life.

The LMSQ is the first questionnaire of its kind that is freely available in German. It approaches the need for methods to assess therapy satisfaction. It could thereby help to facilitate research in the field as well as daily patient care.

The LMSQ positively correlates with medication adherence. This correlation has been demonstrated in other studies using other questionnaires for the measurement of therapy satisfaction [35]. It also correlates with various dimensions of health literacy. Similar observations have also been made with other questionnaires for therapy satisfaction [36].

### 4.2. Limitations

The presented data were only assessed in patients with SSc. This impedes the generalizability of the results to other patient collectives with other medical issues. Furthermore, the questionnaire was only tested in a single center. A multi-center study could provide differing results. This includes potential cultural differences. These could be addressed by performing a study in regions culturally different from Germany. Additionally, this study was conducted using the German version of the LMSQ. The English version presented in Table 1 might show different psychometric properties than the German one. Moreover, the COVID-19 pandemic and its consequences that were close to its peak during the data collection could have played a role in patient recruitment. It seems plausible that patients with a high degree of anxiety could have avoided visiting the hospital due to fear of contracting the virus of and therefore might be underrepresented in our collective [37,38]. This holds especially true considering the immunosuppressive nature of the SSc therapy regimens. Fortunately, sampling issues only play a minor role in studies such as this one since the main goal is to empirically examine the content and measurement dimensions that underpin the theoretical construct underlying the questionnaire [10]. The item-item covariance structure can be assumed to be fairly consistent, even with moderate sampling bias. Studies in larger and more diverse patient collectives would increase the external validity and should therefore be conducted.

This includes two dimensions of further external validation. Firstly, studies in similar collectives to others already extensively analyzed in the field of therapy satisfaction research could help compare the LMSQ to other established questionnaires and thereby elucidate the differences in psychometric properties such as reliability and factor covariance. This would especially be useful to clarify the indications for several questionnaires in research as well as in clinical practice. Diseases with extensive research in the field of therapy satisfaction include diabetes, hypertension, and chronic obstructive pulmonary disease [39,40,41]. These diseases share high prevalence in the general population. Therefore, elucidating the role of therapy satisfaction in these disease entities is of high urgency.

Secondly, further studies for the quantification of therapy satisfaction using the LMSQ could unfold the topic in less common diseases in which therapy satisfaction so far has only played a minor role.

The factor loadings of the LMSQ were only moderately high with values as low as 0.318. This might impede the differentiation between the dimensions of therapy satisfaction represented in the various subscales of the LMSQ. However, all factors were considered statistically significant (*p* < 0.001). Moreover, the overall model of the confirmatory factor analysis displayed excellent fit.

### 4.3. The Future of Therapy Satisfaction in Medicine

Currently, the assessment of therapy satisfaction is far from becoming clinical routine. Therapy satisfaction is rarely considered in therapy decisions. Even when it is considered, it is often not assessed by appropriate methods. Research data on therapy satisfaction is scarce for the majority of diseases and drugs. However, in an effort to shift the clinical practice towards a personalized medicine approach, therapy satisfaction should be tackled with high priority. Questionnaires such as the LMSQ facilitate a quick assessment of therapy satisfaction and its dimensions and thereby enable healthcare providers to shape their therapy according to the patients’ preferences. Given its quick application and non-necessity of medical personnel to complete, it does not bind many valuable human resources. Digitalization tools could make the process more efficient and further decrease the need for human resources [42]. Combining the LMSQ with other PRO-related questionnaires could help adapt therapy approaches specifically to patients.

Science on the application of personalized medicine tended to concentrate on molecular analyses, neglecting the role of therapy satisfaction for the long-term adherence to individual therapy decisions, especially in chronic diseases. Therefore, it is an urgent need to add PROs assessing therapy satisfaction to conventional strategies in personalized medicine. Such a holistic approach might be the key to improve upon not only clinical outcome but also upon other outcome criteria that have so far been treated as secondary.

## 5. Conclusions

The LMSQ is an easily applicable tool for the measurement of therapy satisfaction and its dimensions. Reliability and internal validity have been proven by applying multiple adequate statistical methods. External validity, however, remains unclear; therefore, a validation study in a more diverse patient collective should be conducted. The questionnaire lays the groundwork for further research in the field of therapy satisfaction. The role of therapy satisfaction in clinical practice as well as in research is currently underappreciated. It should be at the forefront of the mind of any healthcare worker when dealing with patients.

## Figures and Tables

**Table 1:** Lübeck Medication Satisfaction Questionnaire.

	Statement	1	2	3	4
LMSQ_1	My medication schedule suits me well.				
LMSQ_2	I feel restricted in my everyday activities due to the side effects of my medication. *				
LMSQ_3	My medication is very convenient to take.				
LMSQ_4	Overall, I am satisfied with my treatment.				
LMSQ_5	My symptoms are being alleviated by my medication.				
LMSQ_6	I feel like my physician is educating me properly about my disease.				
LMSQ_7	I am content with the taste and size of my medications.				
LMSQ_8	The advantages and disadvantages of the treatment options were explained to me by my physician in detail.				
LMSQ_9	I am unable to perform as much physical activity as before due to the side effects of my medication. *				
LMSQ_10	My medication helps me perform personal hygiene tasks (brushing my teeth, taking a shower etc.).				
LMSQ_11	Prior to my treatment, I felt worse than now.				
LMSQ_12	The medication helps me get through my everyday life.				
LMSQ_13	My physician has educated me about the best treatment option.				
LMSQ_14	I am content with the time passing until my medication starts to work.				
LMSQ_15	I am happy with my treatment.				
LMSQ_16	Thanks to the medication, I can participate in leisure activities.				
LMSQ_17	I cannot enjoy my leisure time as much anymore due to the side effects of my medication. *				
LMSQ_18	I intend to continue my treatment.				

The questionnaire consists of 18 statements (LMSQ_1 to LMSQ_18), each rated on a four-point Likert scale ranging from one to four. 1 = I strongly disagree, 2 = I disagree, 3 = I agree, 4 = I strongly agree. Statements LMSQ_2, LMSQ_9, and LMSQ_17 are marked with an asterisk indicating that they were phrased negatively and therefore need to be inverted before the evaluation.

**Table 2:** Demographic characteristics of the participants.

Demographics	
Age ± SD	56.3 ± 13.9
Female (%)	64 (75.3)
Native German Speakers (%)	84 (98.8)
Migration Background (%)	9 (10.6)
Christian (%)	54 (63.6)
Muslim (%)	3 (3.5)
Nonreligious (%)	28 (32.9)
Primary Education (%)	2 (2.4)
Secondary Education (%)	67 (78.8)
Tertiary Education (%)	16 (18.8)
Employed (%)	34 (40)
Unemployed (%)	2 (2.4)
Retired (%)	46 (54.1)
In Education (%)	3 (3.5)
Household members ± SD	2.3 ± 1.1
Disease Duration in months ± SD	123 ± 101
SHAQ score ± SD	1.6 ± 0.6

Abbreviation: SD = standard deviation.

**Table 3:** Descriptives of the LMSQ and its subscales.

Descriptives							
	LMSQ	Side Effects	Effectivity	Practicability	Daily Life	Healthcare Workers	General Satisfaction
N	82	83	82	84	83	84	83
Missing	4	3	4	2	3	2	3
Mean	3.17	2.88	3.17	3.27	2.98	3.41	3.26
Median	3.11	3	3.33	3.17	3	3.33	3.33
Standard deviation	0.37	0.64	0.58	0.52	0.57	0.48	0.47
Minimum	2.33	1.33	1.67	1.67	1	2	2
Maximum	3.94	4	4	4	4	4	4

**Table 4:** Kaiser-Meyer-Olkin test of the LMSQ.

KMO Measure of Sampling Adequacy	
	MSA
Overall	0.811
LMSQ_1	0.662
LMSQ_2	0.672
LMSQ_3	0.703
LMSQ_4	0.842
LMSQ_5	0.834
LMSQ_6	0.772
LMSQ_7	0.867
LMSQ_8	0.827
LMSQ_9	0.711
LMSQ_10	0.849
LMSQ_11	0.765
LMSQ_12	0.836
LMSQ_13	0.786
LMSQ_14	0.849
LMSQ_15	0.915
LMSQ_16	0.829
LMSQ_17	0.784
LMSQ_18	0.819

Abbreviations: KMO = Kaiser-Meyer-Olkin; MSA = measure of sampling adequacy.

**Table 5:** Confirmatory Factor Analysis (Factor Loadings) of the LMSQ.

Factor Loadings					
Factor	Indicator	Estimate	SE	Z	*p*
Side effects	LMSQ_9	0.492	0.1063	4.63	< 0.001
	LMSQ_17	0.513	0.1001	5.13	< 0.001
	LMSQ_2	0.521	0.1139	4.57	< 0.001
Effectivity	LMSQ_14	0.532	0.0628	8.48	<0.001
	LMSQ_11	0.408	0.0827	4.93	<0.001
	LMSQ_5	0.581	0.065	8.92	<0.001
Practicability	LMSQ_3	0.547	0.0993	5.51	<0.001
	LMSQ_7	0.359	0.0903	3.97	<0.001
	LMSQ_1	0.318	0.0869	3.66	<0.001
Daily life	LMSQ_12	0.505	0.0638	7.92	<0.001
	LMSQ_16	0.374	0.0689	5.43	< 0.001
	LMSQ_10	0.584	0.0845	6.91	<0.001
Healthcare workers	LMSQ_13	0.5	0.054	9.25	<0.001
	LMSQ_8	0.352	0.0593	5.93	< 0.001
	LMSQ_6	0.43	0.0544	7.9	<0.001
General Satisfaction	LMSQ_18	0.318	0.0556	5.72	<0.001
	LMSQ_15	0.49	0.0764	6.42	< 0.001
	LMSQ_4	0.354	0.0571	6.19	<0.001

Abbreviations: SE = standard error.

**Table 6:** Additional Measures of Fitness of the LMSQ.

Fit Measures							
				RMSEA 90% CI			
CFI	TLI	SRMR	RMSEA	Lower	Upper	AIC	BIC
0.917	0.895	0.0712	0.0656	0.0372	0.0896	2664	2832

Abbreviations: CFI = Comparative fit index, TLI = Tucker-Lewis-Index, SRMR = Standardized root mean square residual, RMSEA = Root mean square error of approximation, CI = Confidence interval, AIC = Akaike information criterion, BIC = Bayesian information criterion.

**Table 7:** Factor Estimates of the LMSQ.

Factor Covariances					
		Estimate	SE	Z	*p*
Side effects	Side effects	1 ^â^			
	Effectivity	0.443	0.133	3.332	<0.001
	Practicability	0.526	0.1436	3.663	< 0.001
	Daily life	0.257	0.1516	1.697	0.09
	Healthcare workers	0.396	0.1307	3.026	0.002
	General satisfaction	0.566	0.1374	4.122	<0.001
Effectivity	Effectivity	1 ^â^			
	Practicability	0.349	0.1404	2.487	0.013
	Daily life	0.897	0.0598	15.012	<0.001
	Healthcare workers	0.555	0.0979	5.675	<0.001
	General satisfaction	0.946	0.0659	14.355	< 0.001
Practicability	Practicability	1 ^â^			
	Daily life	0.151	0.1612	0.935	0.35
	Healthcare workers	0.179	0.1557	1.15	0.25
	General satisfaction	0.584	0.1394	4.192	< 0.001
Daily life	Daily life	1 ^â^			
	Healthcare workers	0.624	0.0954	6.54	<0.001
	General satisfaction	0.729	0.1031	7.07	< 0.001
Healthcare workers	Healthcare workers	1 ^â^			
	General satisfaction	0.623	0.1088	5.726	< 0.001
General satisfaction	General satisfaction	1 ^â^			

^â^ fixed parameter, Abbreviations: SE = standard error.

**Table 8:** Correlation between the LMSQ score and HLQ subscores (controlling for age, SHAQ score, and time since the disease onset).

	Feeling Understood	Information	Managing	Social Support	Appraisal	Engagement	Navigating	Finding	Understanding
r	0.518	0.458	0.338	0.422	0.12	0.416	0.392	0.331	0.353
*p*	<0.001	<0.001	0.004	<0.001	0.324	<0.001	0.001	0.005	0.003

## Data Availability

The datasets generated and/or analyzed during the current study are available from the corresponding author on reasonable request.

## Supplementary Materials

The following supporting information can be downloaded at: https://www.mdpi.com/article/10.3390/jpm13030505/s1, Table S1: LMSQ—German Version (Lübecker Fragebogen zur Therapiezufriedenheit).

## References

[1] Di Sanzo M., Cipolloni L., Borro M., La Russa R., Santurro A., Scopetti M., Simmaco M., Frati P. (2017). Clinical Applications of Personalized Medicine: A New Paradigm and Challenge. Curr. Pharm. Biotechnol..

[2] Schork N.J. (2015). Personalized medicine: Time for one-person trials. Nature.

[3] Goetz L.H., Schork N.J. (2018). Personalized Medicine: Motivation, Challenges and Progress. Fertil. Steril..

[4] McCabe R., Healey P.G.T. (2018). Miscommunication in Doctor-Patient Communication. Top. Cogn. Sci..

[5] Shikiar R., Rentz A.M. (2004). Satisfaction with medication: An overview of conceptual, methodologic, and regulatory issues. Value Health J. Int. Soc. Pharm. Outcomes Res..

[6] Barbosa C.D., Balp M.M., Kulich K., Germain N., Rofail D. (2012). A literature review to explore the link between treatment satisfaction and adherence, compliance, and persistence. Patient Prefer. Adherence.

[7] Krauss P., Reinartz F., Sonnleitner C., Vazan M., Ringel F., Meyer B., Meyer H.S. (2022). The Relation of Patient Expectations, Satisfaction, and Outcome in Surgery of the Cervical Spine: A Prospective Study. Spine.

[8] Sidani S., Epstein D.R., Fox M., Collins L. (2018). The contribution of participant, treatment, and outcome factors to treatment satisfaction. Res. Nurs. Health.

[9] Desmet M., Van Nieuwenhove K., De Smet M., Meganck R., Deeren B., Van Huele I., Decock E., Raemdonck E., Cornelis S., Truijens F. (2021). What too strict a method obscures about the validity of outcome measures. Psychother. Res. J. Soc. Psychother. Res..

[10] Atkinson M.J., Sinha A., Hass S.L., Colman S.S., Kumar R.N., Brod M., Rowland C.R. (2004). Validation of a general measure of treatment satisfaction, the Treatment Satisfaction Questionnaire for Medication (TSQM), using a national panel study of chronic disease. Health Qual. Life Outcomes.

[11] Atkinson M.J., Kumar R., Cappelleri J.C., Hass S.L. (2005). Hierarchical construct validity of the treatment satisfaction questionnaire for medication (TSQM version II) among outpatient pharmacy consumers. Value Health J. Int. Soc. Pharm. Outcomes Res..

[12] Bharmal M., Payne K., Atkinson M.J., Desrosiers M.P., Morisky D.E., Gemmen E. (2009). Validation of an abbreviated Treatment Satisfaction Questionnaire for Medication (TSQM-9) among patients on antihypertensive medications. Health Qual. Life Outcomes.

[13] Rauthmann J.F., Sherman R.A., Nave C.S., Funder D.C. (2015). Personality-driven situation experience, contact, and construal: How people’s personality traits predict characteristics of their situations in daily life. J. Res. Personal..

[14] Bleidorn W., Hopwood C.J., Lucas R.E. (2018). Life Events and Personality Trait Change. J. Personal..

[15] Denissen J.J.A., Luhmann M., Chung J.M., Bleidorn W. (2019). Transactions between life events and personality traits across the adult lifespan. J. Personal. Soc. Psychol..

[16] Revicki D. (2004). Patient assessment of treatment satisfaction: Methods and practical issues. Gut.

[17] Parmar M., Maturi B., Dutt J.M., Phate H. Sentiment Analysis on Interview Transcripts: An application of NLP for Quantitative Analysis. Proceedings of the 2018 International Conference on Advances in Computing, Communications and Informatics (ICACCI).

[18] Bell K., Fahmy E., Gordon D. (2016). Quantitative conversations: The importance of developing rapport in standardised interviewing. Qual. Quant..

[19] Park H.Y., Seo S.A., Yoo H., Lee K. (2018). Medication adherence and beliefs about medication in elderly patients living alone with chronic diseases. Patient Prefer. Adherence.

[20] Lorusso D., Bria E., Costantini A., Di Maio M., Rosti G., Mancuso A. (2017). Patients’ perception of chemotherapy side effects: Expectations, doctor-patient communication and impact on quality of life—An Italian survey. Eur. J. Cancer Care.

[21] Jneid S., Jabbour H., Hajj A., Sarkis A., Licha H., Hallit S., Khabbaz L.R. (2018). Quality of Life and Its Association with Treatment Satisfaction, Adherence to Medication, and Trust in Physician Among Patients with Hypertension: A Cross-Sectional Designed Study. J. Cardiovasc. Pharmacol. Ther..

[22] Ryu E., Takahashi P.Y., Olson J.E., Hathcock M.A., Novotny P.J., Pathak J., Bielinski S.J., Cerhan J.R., Sloan J.A. (2015). Quantifying the importance of disease burden on perceived general health and depressive symptoms in patients within the Mayo Clinic Biobank. Health Qual. Life Outcomes.

[23] Manzoor F., Wei L., Hussain A., Asif M., Shah S.I.A. (2019). Patient Satisfaction with Health Care Services; An Application of Physician’s Behavior as a Moderator. Int. J. Environ. Res. Public Health.

[24] Hall W.J., Chapman M.V., Lee K.M., Merino Y.M., Thomas T.W., Payne B.K., Eng E., Day S.H., Coyne-Beasley T. (2015). Implicit Racial/Ethnic Bias Among Health Care Professionals and Its Influence on Health Care Outcomes: A Systematic Review. Am. J. Public Health.

[25] Grassi L., Caruso R., Costantini A. (2015). Communication with patients suffering from serious physical illness. Adv. Psychosom. Med..

[26] Matrisch L., Graßhoff H., Müller A., Schinke S., Riemekasten G. (2022). Therapy satisfaction and health literacy are key factors to improve medication adherence in systemic sclerosis. Scand. J. Rheumatol..

[27] Van den Hoogen F., Khanna D., Fransen J., Johnson S.R., Baron M., Tyndall A., Matucci-Cerinic M., Naden R.P., Medsger T.A., Carreira P.E. (2013). 2013 classification criteria for systemic sclerosis: An American college of rheumatology/European league against rheumatism collaborative initiative. Ann. Rheum. Dis..

[28] Johnson S.R., Hawker G.A., Davis A.M. (2005). The health assessment questionnaire disability index and scleroderma health assessment questionnaire in scleroderma trials: An evaluation of their measurement properties. Arthritis Care Res. Off. J. Am. Coll. Rheumatol..

[29] De Klerk E., van der Heijde D., Landewé R., van der Tempel H., van der Linden S. (2003). The compliance-questionnaire-rheumatology compared with electronic medication event monitoring: A validation study. J. Rheumatol..

[30] Osborne R.H., Batterham R.W., Elsworth G.R., Hawkins M., Buchbinder R. (2013). The grounded psychometric development and initial validation of the Health Literacy Questionnaire (HLQ). BMC Public Health.

[31] Beaton D.E., Bombardier C., Guillemin F., Ferraz M.B. (2000). Guidelines for the process of cross-cultural adaptation of self-report measures. Spine.

[32] Kaiser H. (1974). An Index of Factorial Simplicity. Psychometrika.

[33] Leonard K.V., Robertson C., Bhowmick A., Herbert L.B. (2020). Perceived Treatment Satisfaction and Effectiveness Facilitators Among Patients With Chronic Health Conditions: A Self-Reported Survey. Interact. J. Med. Res..

[34] Ruiz M.A., Pardo A., Rejas J., Soto J., Villasante F., Aranguren J.L. (2008). Development and Validation of the “Treatment Satisfaction with Medicines Questionnaire” (SATMED-Q)^©^. Value Health.

[35] Unni E., Bae S. (2022). Exploring a New Theoretical Model to Explain the Behavior of Medication Adherence. Pharmacy.

[36] Altin S.V., Stock S. (2016). The impact of health literacy, patient-centered communication and shared decision-making on patients’ satisfaction with care received in German primary care practices. BMC Health Serv. Res..

[37] Czeisler M.É., Kennedy J.L., Wiley J.F., Facer-Childs E.R., Robbins R., Barger L.K., Czeisler C.A., Rajaratnam S.M., Howard M.E. (2021). Delay or avoidance of routine, urgent and emergency medical care due to concerns about COVID-19 in a region with low COVID-19 prevalence: Victoria, Australia. Respirology.

[38] Kernder A., Filla T., de Groot K., Hellmich B., Holle J., Lamprecht P., Moosig F., Ruffer N., Specker C., Vordenbäumen S. (2022). COVID-19 pandemic impairs medical care of vasculitis patients in Germany: Results of a national patient survey. Front. Med..

[39] Mita T., Katakami N., Takahara M., Kawashima M., Wada F., Akiyama H., Morita N., Kidani Y., Yajima T., Shimomura I. (2022). Changes in Treatment Satisfaction Over 3 Years in Patients with Type 2 Diabetes after Initiating Second-line Treatment. J. Clin. Endocrinol. Metab..

[40] López-Torres López J., Blázquez Abellán G., López-Torres Hidalgo M.R., Milián García R.M., López Martínez C. (2019). Evaluation of satisfaction with pharmacological treatment in people with hypertension. Rev. Esp. Salud Publica.

[41] Contoli M., Rogliani P., Di Marco F., Braido F., Corsico A.G., Amici C.A., Piro R., Sarzani R., Lessi P., Scognamillo C. (2019). Satisfaction with chronic obstructive pulmonary disease treatment: Results from a multicenter, observational study. Ther. Adv. Respir. Dis..

[42] Benze G., Nauck F., Alt-Epping B., Gianni G., Bauknecht T., Ettl J., Munte A., Kretzschmar L., Gaertner J. (2019). PROutine: A feasibility study assessing surveillance of electronic patient reported outcomes and adherence via smartphone app in advanced cancer. Ann. Palliat. Med..

