# Supplementary material for: The Lübeck Medication Satisfaction Questionnaire—A Novel Measurement Tool for Therapy Satisfaction
Source: J Pers Med. 2023 Mar 10;13(3):505. doi: 10.3390/jpm13030505 (PMC10058402; doi:10.3390/jpm13030505)
Supplement: Supplementary file 1 [file jpm-13-00505-s001.zip › jpm-2197240-supplementary.pdf]

Supplement

Supplementary Table S1: LMSQ – German Version (Lübecker Fragebogen zur Therapiezufriedenheit)

	**Aussage**	**1**	**2**	**3**	**4**
**LMSQ_1**	Mir passt der Zeitplan, nach dem ich meine Medikamente nehme, sehr gut.				
**LMSQ_2**	Ich fühle mich in meinen Alltagsaktivitäten durch die Nebenwirkungen meiner Medikamente eingeschränkt.*				
**LMSQ_3**	Meine Medikamente sind sehr bequem zu nehmen.				
**LMSQ_4**	Meine Behandlung stellt mich im Großen und Ganzen zufrieden.				
**LMSQ_5**	Meine Symptome werden durch meine Medikamente gelindert.				
**LMSQ_6**	Ich fühle mich durch meinen Arzt/meine Ärztin gut über meine Erkrankung aufgeklärt.				
**LMSQ_7**	Ich bin mit dem Geschmack und der Größe meiner Medikamente zufrieden.				
**LMSQ_8**	Mein Arzt/meine Ärztin hat mir die Vor- und Nachteile der Behandlungsmöglichkeiten genau erläutert.				
**LMSQ_9**	Ich kann durch die Nebenwirkungen meiner Medikamente weniger Sport machen.*				
**LMSQ_10**	Durch meine Medikamente fällt es mir leichter, Körperpflege (Zähne putzen, duschen usw.) zu betreiben.				
**LMSQ_11**	Vor Beginn der Behandlung habe ich mich schlechter gefühlt als jetzt.				
**LMSQ_12**	Die Medikamente helfen mir, durch meinen Alltag zu kommen.				
**LMSQ_13**	Mein Arzt/Meine Ärztin hat mich über die beste Art der Behandlung aufgeklärt.				
**LMSQ_14**	Ich bin zufrieden mit der Länge der Zeit, die vergeht, bis meine Medikamente wirken.				
**LMSQ_15**	Ich bin glücklich mit meiner Behandlung.				
**LMSQ_16**	Dank der Medikamente kann ich an Freizeitaktivitäten teilnehmen.				

**LMSQ_17**	Ich kann durch die Nebenwirkungen der Medikamente meine Freizeit nicht mehr so sehr genießen.*				
**LMSQ_18**	Ich habe vor, meine Behandlung fortzusetzen.				

Supplementary Table S1. German version of the Lübeck Medication Satisfaction Questionnaire. The questionnaire consists of 18 statements (LMSQ_1 to LMSQ_18) each rated on a four-point Likert scale ranging from one to four. 1=stimme gar nicht zu, 2=stimme nicht zu, 3=stimme zu, 4=stimme voll zu. Statements LMSQ_2, LMSQ_9 and LMSQ_17 are marked with an asterisk indicating that they were phrased negatively and therefore need to be inverted before the evaluation.
